# Supplementary material for: Adherence to the 2017 French dietary guidelines and adult weight gain: A cohort study
Source: PLoS Med. 2019 Dec 30;16(12):e1003007. doi: 10.1371/journal.pmed.1003007 (PMC6936788; doi:10.1371/journal.pmed.1003007)
Supplement: S1 Table — (DOCX) [file pmed.1003007.s002.docx]

S1 Table – Characteristics of the participants by quintile of mPNNS-GS1, NutriNet-Santé study, N=54,089 ^a^.

|  |  | **Total** | **Q1** | **Q2** | **Q3** | **Q4** | **Q5** |
| --- | --- | --- | --- | --- | --- | --- | --- |
| **PNNS-GS2 mean (sd)** |  | 1.7 (3.3) | -1.2 (2.9) | 0.5 (2.8) | 1.5 (2.8) | 2.7 (2.7) | 4.4 (2.6) |
| **sPNNS-GS2 mean(sd)** |  | 1.9 (3.5) | -1.3 (3.1) | 0.6 (3) | 1.6 (2.9) | 2.9 (2.7) | 4.8 (2.6) |
| **mPNNS-GS1 mean(sd)** |  | 8.1 (1.6) | 5.7 (0.8) | 7.1 (0.3) | 8 (0.2) | 8.9 (0.3) | 10.3 (0.7) |
| **Age at inclusion (years) ^b^** |  | 47.1 (14.1) | 41 (13.8) | 44.1 (14.1) | 47 (13.8) | 49.4 (13.5) | 52.4 (12.8) |
| **Weight measurements ^b^** |  | 11.4 (4.7) | 10.5 (4.7) | 10.9 (4.7) | 11.5 (4.7) | 11.8 (4.6) | 12.1 (4.7) |
| **Height (cm) ^b^** |  | 166.5 (8.2) | 167.3 (8.3) | 166.8 (8.2) | 167.2 (8.4) | 166 (8) | 165.3 (7.8) |
| **Body Mass Index (kg/m²) ^b^** |  | 23.9 (4.4) | 23.5 (4.7) | 23.9 (4.6) | 24.1 (4.4) | 23.9 (4.3) | 23.9 (4.3) |
| **Energy intake without alcohol (kcal/d) ^b^** |  | 1821.5 (441.3) | 2006.1 (530.7) | 1844.2 (445.4) | 1819.4 (427.2) | 1757.1 (395.2) | 1724.9 (364.2) |
| **Ethanol consumption (g/d) ^b^** |  | 8.3 (11.5) | 11.1 (15.2) | 9.4 (13.1) | 8.6 (11) | 7.3 (9.4) | 5.7 (7.5) |
| **Sex ^c^** |  |  |  |  |  |  |  |
| **Female** |  | 76.1% | 76.2% | 75.9% | 71.1% | 77.5% | 79.3% |
| **Male** |  | 23.9% | 23.8% | 24.1% | 28.9% | 22.5% | 20.7% |
| **Education ^d^** |  |  |  |  |  |  |  |
| **Primary** |  | 1% | 1.2% | 1.1% | 1% | 0.9% | 0.9% |
| **Secondary** |  | 35.5% | 36.2% | 35.5% | 34.9% | 35.2% | 36% |
| **University** |  | 63.5% | 62.6% | 63.4% | 64.2% | 63.9% | 63.1% |
| **Occupational category ^e^** |  |  |  |  |  |  |  |
| **Farmers / self-employed** |  | 1.9% | 2.4% | 2% | 2.2% | 1.6% | 1.5% |
| **Managerial staff** |  | 23% | 20.9% | 22.9% | 25% | 23.2% | 22.4% |
| **Employees** |  | 16.1% | 20.6% | 18.6% | 15.2% | 14.8% | 12.1% |
| **Students** |  | 5.9% | 10.3% | 7.6% | 5.3% | 4% | 3.2% |
| **Manual workers** |  | 1% | 1.7% | 1.5% | 1% | 0.7% | 0.6% |
| **Intermediates professions** |  | 17.1% | 18.2% | 17.7% | 17.4% | 17.1% | 15.5% |
| **Retired** |  | 23.8% | 12.5% | 18% | 23.1% | 27.7% | 34.6% |
| **Unemployed** |  | 11.2% | 13.3% | 11.6% | 10.8% | 10.7% | 10.1% |
| **Income ^d^** |  |  |  |  |  |  |  |
| **≤1800 €/cu ^f^** |  | 43.3% | 55.5% | 48.8% | 42.6% | 39.1% | 34% |
| **1800 - 2700 €/cu** |  | 27% | 24.3% | 25.9% | 26.9% | 27.6% | 29.5% |
| **>2700 €/cu** |  | 29.7% | 20.2% | 25.3% | 30.4% | 33.4% | 36.5% |
| **Physical activity ^d^** |  |  |  |  |  |  |  |
| **[0-30 [ min/day** |  | 25.2% | 34.7% | 29% | 25.2% | 21.8% | 17.9% |
| **[30-60 [ min/ day** |  | 23.9% | 23.4% | 25.2% | 24.1% | 23.5% | 23.3% |
| **≥ 60 min/ day** |  | 50.9% | 41.9% | 45.8% | 50.6% | 54.7% | 58.8% |
| **Smoking ^d^** |  |  |  |  |  |  |  |
| **Non smokers** |  | 49.9% | 49% | 49.7% | 49.1% | 49.6% | 52% |
| **Former smokers** |  | 36.5% | 29.9% | 33.7% | 37.4% | 39.4% | 40.2% |
| **Smokers** |  | 13.6% | 21.1% | 16.6% | 13.5% | 11% | 7.8% |
| **Living status ^c^** |  |  |  |  |  |  |  |
| **Living alone** |  | 27.6% | 30% | 28.2% | 26.1% | 26.1% | 28% |
| **Cohabiting** |  | 72.4% | 70% | 71.8% | 73.9% | 73.8% | 72% |

^a^ Values are percentages or mean (standard deviation) as appropriate. All p-values were <0.001.

^b^ Linear contrast trend test

^c^ Cochran-Armitage trend test

^d^ Spearman correlation test for ordinal variables

^e^ Pearson Chi Square association test

^f^ cu = consumption unit
