# Supplementary material for: Adherence to the 2017 French dietary guidelines and adult weight gain: A cohort study
Source: PLoS Med. 2019 Dec 30;16(12):e1003007. doi: 10.1371/journal.pmed.1003007 (PMC6936788; doi:10.1371/journal.pmed.1003007)
Supplement: S2 Table — (DOCX) [file pmed.1003007.s003.docx]

S2 Table – Comparison of baseline characteristics of the participants in the whole NutriNet-Santé study (N=138,014) and in our selected population (N=54,089) ^a^.

|  |  | **Selected population** | **Whole population** | **p** |
| --- | --- | --- | --- | --- |
| **Age at inclusion (years) ^b^** |  | 47.1 (14.1) | 42.3 (14.7) | <0.001 |
| **Body Mass Index (kg/m²) ^b^** |  | 23.9 (4.4) | 24 (4.8) | <0.001 |
| **Physical activity (METs) ^b^** |  | 2869 (2769) | 2735 (2697) | <0.001 |
| **Sex ^c^** |  |  |  | <0.001 |
| **Female** |  | 76.1% | 77.7% |  |
| **Male** |  | 23.9% | 22.3% |  |
| **Education ^c^** |  |  |  | <0.001 |
| **Primary** |  | 1.0% | 1.4% |  |
| **Secondary** |  | 35.5% | 36.9% |  |
| **University** |  | 63.5% | 61.7% |  |
| **Occupational category ^c^** |  |  |  | <0.001 |
| **Farmers / self-employed** |  | 1.9% | 2.2% |  |
| **Managerial staff** |  | 23.0% | 22.2% |  |
| **Employees** |  | 16.1% | 19.4% |  |
| **Students** |  | 5.9% | 9.2% |  |
| **Manual workers** |  | 1.0% | 1.5% |  |
| **Intermediates professions** |  | 17.1% | 16.4% |  |
| **Retired** |  | 23.8% | 16.6% |  |
| **Unemployed** |  | 11.2% | 12.7% |  |
| **Income ^c^** |  |  |  | <0.001 |
| **≤1800 €/cu** |  | 43.3% | 49.7% |  |
| **1800 - 2700 €/cu** |  | 27.0% | 25.3% |  |
| **>2700 €/cu** |  | 29.7% | 25.0% |  |
| **Smoking ^c^** |  |  |  | <0.001 |
| **Non-smokers** |  | 49.9% | 51.6% |  |
| **Former smokers** |  | 36.5% | 34.5% |  |
| **Smokers** |  | 13.6% | 13.9% |  |
| **Living status ^c^** |  |  |  | <0.001 |
| **Living alone** |  | 27.6% | 29.9% |  |
| **Cohabiting** |  | 72.4% | 70.1% |  |

Abbreviations: cu = consumption unit, METs = Metabolic Equivalents

^a^ Values are percentages or mean (standard deviation) as appropriate.

^b^ Two Sample t-test

^c^ Pearson Chi Square association test
