# Supplementary material for: Adherence to the 2017 French dietary guidelines and adult weight gain: A cohort study
Source: PLoS Med. 2019 Dec 30;16(12):e1003007. doi: 10.1371/journal.pmed.1003007 (PMC6936788; doi:10.1371/journal.pmed.1003007)
Supplement: S4 Table — (DOCX) [file pmed.1003007.s005.docx]

S4 Table – Longitudinal evolution of log(BMI) as a function of sPNNS-GS2, NutriNet-Santé study ^a^

|  |  | **sPNNS-GS2** |  |
| --- | --- | --- | --- |
|  |  | **β [95% IC]** ^a^ | **p** ^d^ |
| **m0** ^b^ |  |  |  |
| Score (1 sd) |  | -0.033 [-0.035;-0.031] | <0.001 |
| Time (years) |  | 0.0026 [0.0024;0.0027] | <0.001 |
| Score × Time |  | -0.00067 [-0.00082;-0.00053] | <0.001 |
| **m1** ^c^ |  |  |  |
| Score (1 sd) |  | -0.030 [-0.032;-0.028] | <0.001 |
| Time (years) |  | 0.0026 [0.0024;0.0027] | <0.001 |
| Score × Time |  | -0.00067 [-0.00082;-0.00053] | <0.001 |

^a^ Coefficients β were computed using a linear multilevel mixed model expressing the relationship between log(BMI) and sPNNS-GS2 (expressed as number of standard deviations) and time (in years). Logarithm of BMI was used to increase normality and model’s residual fitness.

Coefficient for score represents the association of FBGD with baseline BMI, coefficient for time represents the mean evolution of BMI over time and coefficient for the interaction term represents the association of FBGD with evolution of BMI over time.

As BMI was log-transformed, interpretation of the interaction term is less intuitive since additive effects on log(BMI) becomes multiplicative on BMI, e.g. for an increase of 2 SD of score and 5 years, BMI is multiplied by$\exp\left( 2\beta_{\mathrm{score}}+5\beta_{\mathrm{time}}+5{\times2\times\beta}_{score\times time} \right).$

^b^ m0 is the base model, adjusted for sex, energy intake without alcohol and number of completed 24h dietary records,

with second and third order interaction terms between sex, time and dietary score

^c^ m1 is the full model, further adjusted for height, month of inclusion, physical activity, socioeconomic level, smoking status, educational level, monthly income and cohabiting status, with second and third order interaction terms between sex, time and dietary score

^d^ p-values were computed using a Wald test for coefficient nullity
