# Supplementary material for: Adherence to the 2017 French dietary guidelines and adult weight gain: A cohort study
Source: PLoS Med. 2019 Dec 30;16(12):e1003007. doi: 10.1371/journal.pmed.1003007 (PMC6936788; doi:10.1371/journal.pmed.1003007)
Supplement: S5 Table — (DOCX) [file pmed.1003007.s006.docx]

S5 Table – Prospective association between the PNNS-GS2 and mPNNS-GS and the risk of morbid obesity (BMI>40), NutriNet-Santé study ^a^

|  |  | **PNNSGS-2** |  |  |  | **mPNNS-GS** |  |  |
| --- | --- | --- | --- | --- | --- | --- | --- | --- |
|  |  | **n_sane_/n_case_** | **HR [95% CI]** | **p** ^b^ |  | **n_sane_/n_case_** | **HR [95% CI]** | **p** ^b^ |
| **m0** ^c^ |  |  |  | <0.001 |  |  |  | 0.39 |
| Q1 |  | 9434/113 | 1 |  |  | 9770/64 |  |  |
| Q2 |  | 9482/67 | **0.56 [0.42-0.77]** | 0.0002 |  | 9596/65 | 1.03 [0.73-1.47] | 0.86 |
| Q3 |  | 9447/46 | **0.38 [0.27-0.54]** | <0.001 |  | 10843/80 | 1.07 [0.76-1.51] | 0.68 |
| Q4 |  | 9470/58 | **0.47 [0.34-0.66]** | <0.001 |  | 7372/43 | 0.85 [0.57-1.27] | 0.43 |
| Q5 |  | 9465/31 | **0.25 [0.16-0.37]** | <0.001 |  | 9108/58 | 0.90 [0.62-1.31] | 0.57 |
| 1 point ^e^ |  | 47298/315 | **0.88 [0.85-0.91]** | <0.001 |  | 46689/310 | 0.96 [0.89-1.03] | 0.26 |
| 1 SD ^e^ |  | 47298/315 | **0.64 [0.57-0.73]** | <0.001 |  | 46689/310 | 0.93 [0.83-1.05] | 0.26 |
| **m1** ^d^ |  |  |  | <0.001 |  |  |  | 0.73 |
| Q1 |  | 9434/113 | 1 |  |  | 9770/64 |  |  |
| Q2 |  | 9482/67 | **0.58 [0.43-0.79]** | 0.0005 |  | 9596/65 | 1.13 [0.79-1.61] | 0.51 |
| Q3 |  | 9447/46 | **0.40 [0.28-0.57]** | <0.001 |  | 10843/80 | 1.25 [0.88-1.76] | 0.21 |
| Q4 |  | 9470/58 | **0.51 [0.36-0.71]** | <0.001 |  | 7372/43 | 1.00 [0.67-1.50] | 0.99 |
| Q5 |  | 9465/31 | **0.27 [0.18-0.41]** | <0.001 |  | 9108/58 | 1.12 [0.76-1.65] | 0.57 |
| 1 point ^e^ |  | 47298/315 | **0.89 [0.85-0.92]** | <0.001 |  | 46689/310 | 1.01 [0.93-1.09] | 0.84 |
| 1 SD ^e^ |  | 47298/315 | **0.67 [0.59-0.76]** | <0.001 |  | 46689/310 | 1.01 [0.90-1.14] | 0.84 |

^a^ Bold values are significant

^b^ p-values for whole models are computed using a linear trend test on quintiles’ medians. P-values for coefficients are computed using a Wald test for coefficient nullity.

^c^ m0 is the base model, adjusted for sex, energy intake without alcohol and number of completed 24h dietary records

^d^ m1 is the full model, further adjusted for height, month of inclusion, physical activity, socioeconomic level, smoking status, educational level, monthly income and cohabiting status

^e^ The HR for 1 SD allows the comparison between the two scores, whereas the HR for 1 point gives an “absolute” estimation of the score effect. Yet, caution is advised when interpreting these values with the mPNNS-GS as the linearity hypothesis was not satisfyingly verified.
