# Supplementary material for: Adherence to the 2017 French dietary guidelines and adult weight gain: A cohort study
Source: PLoS Med. 2019 Dec 30;16(12):e1003007. doi: 10.1371/journal.pmed.1003007 (PMC6936788; doi:10.1371/journal.pmed.1003007)
Supplement: S6 Table — (DOCX) [file pmed.1003007.s007.docx]

S6 Table – Prospective association between the sPNNS-GS2 and the risk of overweight and obesity, NutriNet-Santé study ^a^

|  |  | **Overweight** |  |  |  | **Obesity** |  |  |
| --- | --- | --- | --- | --- | --- | --- | --- | --- |
|  |  | **n_sane_/n_case_** | **HR [95% CI]** | **p** ^b^ |  | **n_sane_/n_case_** | **HR [95% CI]** | **p** ^b^ |
| **m0** ^c^ |  |  |  | <0.001 |  |  |  | <0.001 |
| Q1 |  | 5586/969 |  |  |  | 8334/435 |  |  |
| Q2 |  | 5719/834 | **0.78 [0.71-0.86]** | <0.001 |  | 8290/440 | 0.92 [0.80-1.05] | 0.22 |
| Q3 |  | 5717/821 | **0.73 [0.66-0.80]** | <0.001 |  | 8390/368 | **0.71 [0.62-0.82]** | <0.001 |
| Q4 |  | 5850/679 | **0.57 [0.52-0.63]** | <0.001 |  | 8400/316 | **0.57 [0.49-0.67]** | <0.001 |
| Q5 |  | 5957/575 | **0.46 [0.41-0.51]** | <0.001 |  | 8461/253 | **0.43 [0.36-0.50]** | <0.001 |
| 1 point ^e^ |  | 28829/3878 | **0.93 [0.92-0.94]** | <0.001 |  | 41875/1812 | **0.93 [0.92-0.94]** | <0.001 |
| 1 SD ^e^ |  | 28829/3878 | **0.78 [0.75-0.80]** | <0.001 |  | 41875/1812 | **0.77 [0.74-0.81]** | <0.001 |
| **m1** ^d^ |  |  |  | <0.001 |  |  |  | <0.001 |
| Q1 |  | 5586/969 |  |  |  | 8334/435 |  |  |
| Q2 |  | 5719/834 | **0.80 [0.72-0.87]** | <0.001 |  | 8290/440 | 0.95 [0.83-1.09] | 0.44 |
| Q3 |  | 5717/821 | **0.75 [0.68-0.82]** | <0.001 |  | 8390/368 | **0.75 [0.65-0.87]** | <0.001 |
| Q4 |  | 5850/679 | **0.59 [0.54-0.66]** | <0.001 |  | 8400/316 | **0.62 [0.53-0.73]** | <0.001 |
| Q5 |  | 5957/575 | **0.48 [0.43-0.54]** | <0.001 |  | 8461/253 | **0.47 [0.40-0.56]** | <0.001 |
| 1 point ^e^ |  | 28829/3878 | **0.93 [0.92-0.94]** | <0.001 |  | 41875/1812 | **0.94 [0.93-0.95]** | <0.001 |
| 1 SD ^e^ |  | 28829/3878 | **0.79 [0.76-0.82]** | <0.001 |  | 41875/1812 | **0.80 [0.76-0.84]** | <0.001 |

^a^ Bold values are significant

^b^ p-values for whole models are computed using a linear trend test on quintiles’ medians. p-values for coefficients are computed using a Wald test for coefficient nullity.

^c^ m0 is the base model, adjusted for sex, energy intake without alcohol and number of completed 24h dietary records

^d^ m1 is the full model, further adjusted for height, month of inclusion, physical activity, socioeconomic level, smoking status, educational level, monthly income and cohabiting status

^e^ The HR for 1 SD allows the comparison between the two scores, whereas the HR for 1 point gives an “absolute” estimation of the score effect. Yet, caution is advised when interpreting these values with the mPNNS-GS as the linearity hypothesis was not satisfyingly verified.
