# Supplementary material for: Adherence to the 2017 French dietary guidelines and adult weight gain: A cohort study
Source: PLoS Med. 2019 Dec 30;16(12):e1003007. doi: 10.1371/journal.pmed.1003007 (PMC6936788; doi:10.1371/journal.pmed.1003007)
Supplement: S7 Table — (DOCX) [file pmed.1003007.s008.docx]

S7 Table – Prospective association between the PNNS-GS2 and the risk of overweight and obesity on a sub-population for sensitivity analysis, NutriNet-Santé study ^a^

|  |  | **Overweight** |  |  | |  | **Obesity** | |  | |  | |
| --- | --- | --- | --- | --- | --- | --- | --- | --- | --- | --- | --- | --- |
|  |  | **n_sane_/n_case_** | **HR [95% CI]** | **p** ^b^ | |  | **n_sane_/n_case_** | | **HR [95% CI]** | | **p** ^b^ | |
| **m0** ^c^ |  |  |  | <0.0001 | |  |  | |  | | <0.0001 | |
| Q1 |  | 5088/903 | 1 |  |  | 7608/389 | | 1 | |  | |  |
| Q2 |  | 5191/769 | **0.77 [0.70-0.85]** | <0.0001 |  | 7599/376 | | 0.87 [0.75-1.00] | | 0.054 | |  |
| Q3 |  | 5217/752 | **0.71 [0.64-0.79]** | <0.0001 |  | 7691/324 | | **0.69 [0.59-0.80]** | | <0.0001 | |  |
| Q4 |  | 5314/644 | **0.57 [0.52-0.64]** | <0.0001 |  | 7636/277 | | **0.55 [0.47-0.65]** | | <0.0001 | |  |
| Q5 |  | 5418/535 | **0.45 [0.40-0.51]** | <0.0001 |  | 7729/222 | | **0.41 [0.35-0.49]** | | <0.0001 | |  |
| 1 point ^e^ |  | 26228/3603 | **0.92 [0.91-0.93]** | <0.0001 |  | 38263/1588 | | **0.90 [0.89-0.92]** | | <0.0001 | |  |
| 1 SD ^e^ |  | 26228/3603 | **0.75 [0.72-0.78]** | <0.0001 |  | 38263/1588 | | **0.71 [0.67-0.75]** | | <0.0001 | |  |
| **m1** ^d^ |  |  |  | <0.0001 |  |  | |  | | <0.0001 | |  |
| Q1 |  | 5088/903 | 1 |  |  | 7608/389 | | 1 | |  | |  |
| Q2 |  | 5191/769 | **0.79 [0.71-0.87]** | <0.0001 |  | 7599/376 | | 0.90 [0.78-1.04] | | 0.14 | |  |
| Q3 |  | 5217/752 | **0.73 [0.66-0.80]** | <0.0001 |  | 7691/324 | | **0.73 [0.63-0.85]** | | <0.0001 | |  |
| Q4 |  | 5314/644 | **0.60 [0.54-0.66]** | <0.0001 |  | 7636/277 | | **0.60 [0.51-0.71]** | | <0.0001 | |  |
| Q5 |  | 5418/535 | **0.47 [0.42-0.53]** | <0.0001 |  | 7729/222 | | **0.46 [0.38-0.55]** | | <0.0001 | |  |
| 1 point ^e^ |  | 26228/3603 | **0.92 [0.91-0.93]** | <0.0001 |  | 38263/1588 | | **0.91 [0.90-0.93]** | | <0.0001 | |  |
| 1 SD ^e^ |  | 26228/3603 | **0.76 [0.73-0.79]** | <0.0001 |  | 38263/1588 | | **0.74 [0.70-0.78]** | | <0.0001 | |  |

^a^ Subpopulation without cases of incident cancer, bariatric surgery, eating disorder and extreme BMI. Bold values are significant for alpha at 5%.

^b^ p-values are computed using a linear trend test on quintiles’ medians.

^c^ m0 is the base model, adjusted for sex, energy intake without alcohol and number of completed 24h dietary records

^d^ m1 is the full model, further adjusted for height, month of inclusion, physical activity, socioeconomic level, smoking status, educational level, monthly income and cohabiting status

^e^ for modeling of continuous scores, HR is given for 1 point and for 1 SD of each score. The HR for 1 SD allows the comparison between the two scores, whereas the HR for 1 point gives an “absolute” estimation of the score effect.
