# Supplementary material for: Adherence to the 2017 French dietary guidelines and adult weight gain: A cohort study
Source: PLoS Med. 2019 Dec 30;16(12):e1003007. doi: 10.1371/journal.pmed.1003007 (PMC6936788; doi:10.1371/journal.pmed.1003007)
Supplement: S8 Table — (DOCX) [file pmed.1003007.s009.docx]

S8 Table – “Direct comparison” of the predictive value of PNNS-GS2 and AHEI-2010 on the risk of overweight and obesity, NutriNet-Santé study ^a^

|  |  | **Overweight** |  |  |  | **Obesity** |  |  |
| --- | --- | --- | --- | --- | --- | --- | --- | --- |
|  |  | **HR** | **LRT** ^b^ | **Wald** ^c^ |  | **HR** | **LRT** ^b^ | **Wald** ^c^ |
|  |  |  |  | 0.06 |  |  |  | 0.16 |
| PNNS-GS2 |  | 0.83 [0.79-0.87] | <0.001 |  |  | 0.82 [0.76-0.88] | <0.001 |  |
| AHEI-2010 |  | 0.89 [0.85-0.94] | <0.001 |  |  | 0.88 [0.82-0.94] | <0.001 |  |

^a^ Each dietary score was standardized (by dividing by their SD) and considered as a continuous variable in the Cox proportional hazard models. This permits to compare the effect of a dietary score on the outcome, while the other is fixed. Models were adjusted for sex, energy intake without alcohol, number of completed 24h dietary records, height, month of inclusion, physical activity, socioeconomic level, smoking status, educational level, monthly income and cohabiting status. AHEI-2010 is a dietary score based on a comprehensive review of the relevant literature in order to best predict the risk of chronic diseases.

^b^ p-values were computed using a Likelihood Ratio Test measuring the decrease in information when the variable is dropped from the adjusted model

^c^ p-values were computed using a Wald test for coefficient equality between the two dietary scores
